# Specificity Testing for NGT PCR-Based Detection Methods in the Context of the EU GMO Regulations

**DOI:** 10.3390/foods12234298

**Published:** 2023-11-28

**Authors:** Caroline Bedin Zanatta, Aline Martins Hoepers, Rubens Onofre Nodari, Sarah Zanon Agapito-Tenfen

**Affiliations:** 1 Department of Crop Science, Federal University of Santa Catarina, Florianópolis 88034000, Brazil; 2 Climate and Environmental Division, NORCE Norwegian Research AS, 5008 Bergen, Norway

**Keywords:** genetically modified organisms, site-directed nucleases, CRISPR-Cas9, bioinformatics, detection, traceability

## Abstract

The term new genomic techniques (NGTs) is an umbrella term used to describe a variety of techniques that can alter the genetic material of an organism and that have emerged or have been developed since 2001, when the existing genetically modified organism (GMO) legislation was adopted. The analytical framework used to detect GMOs in Europe is an established single harmonized procedure that is mandatory for the authorization of GM food and feed, thus generating a reliable, transparent, and effective labeling scheme for GMO products. However, NGT products can challenge the implementation and enforcement of the current regulatory system in the EU, relating in particular to the detection of NGT products that contain no foreign genetic material. Consequently, the current detection methods might fail to meet the minimum performance requirements. Although existing detection methods may be able to detect and quantify even small alterations in the genome, this does not necessarily confirm the distinction between products resulting from NGTs subject to the GMO legislation and other products. Therefore, this study provides a stepwise approach for the in silico prediction of PCR systems’ specificity by testing a bioinformatics pipeline for amplicon and primer set searches in current genomic databases. In addition, it also empirically tested the PCR system evaluated during the in silico analysis. Two mutant genotypes produced by CRISPR-Cas9 in *Arabidopsis thaliana* were used as a case study. Overall, our results demonstrate that the single PCR system developed for identifying a nucleotide insertion in the grf1-3 genotype has multiple matches in the databases, which do not enable the discrimination of this mutated event. Empirical assays further support this demonstration. In contrast, the second mutated genotype, grf8-61, which contains a -3 bp deletion, did not yield any matches in the sequence variant database. However, the primer sequences were not efficient during the empirical assay. Our approach represents a first step in decision making for analytical methods for NGT detection, identification, and quantification in light of the European labeling regulations.

## 1. Introduction

Genetically modified organisms (GMOs) are subject to regulations in Europe and worldwide through domestic legislation and the Cartagena Protocol on Biosafety (CPB), an international GMO treaty from the United Nations. In Europe, prior to market approval of GMOs and derived food and feed products, GMO event-specific methods for their detection, identification, and quantification need to be in place, according to European Commission (EC) Regulation No 1829/2003 on genetically modified food and feed. The analytical framework to detect GMOs in Europe relies on validated qualitative and quantitative real-time PCR methods, forming part of the single harmonized, time-limited, and transparent procedure for the authorization of GM food and feed in the European Union (EU) [1].

Recent advances in genetic engineering, known as ‘new genomic techniques’ (NGTs), have raised significant concerns regarding the efficacy of current GMO detection methods and their feasibility based on existing regulations. These concerns are supported by the fact that NGT organisms can have small target genomic alterations, involving a few nitrogenous bases that can modify the genotype and/or phenotype for intentional modification [1]. These techniques that allow modifications in the genome are based on site-directed nucleases (SDNs) [2] and encompass different enzymes, such as meganucleases (LAGLIDADG endonucleases; EMNs), zinc finger nucleases (ZNFs), effector transcription factor nucleases (TALENs), and a clustered regularly interspaced short palindromic repeats system (CRISPR-Cas9) [3,4,5,6,7].

In 2018, the Court of Justice of the European Union (CJEU) issued a significant decision stating that gene-editing techniques, including CRISPR-Cas9, should be classified and regulated as GMOs. As a result, any techniques that modify the genetic material of plants or animals using NGTs should be subject to the same regulations applied to GMOs. In this context, the EU has taken significant steps to implement legislation ensuring the traceability of GMO-containing foods and derivatives in the market, providing scientific guidelines and technical documents to empower consumers with informed choices through proper labeling [8,9,10,11].

In the context of detecting NGTs, two main steps are crucial for the development of PCR-based analysis methods: in silico tests and empirical tests. The in silico computational assessments involve the verification of the compatibility of primer sequences and the avoidance of unintended amplifications of other genomic regions or sequences from different organisms. Ensuring the specificity of primers designed for the edited organism is essential, taking into consideration any base differences. PCR relies on the complementarity between primers and the target DNA for successful amplification. The design of specific primers and probes involves predicting secondary structures to avoid interference, such as primer dimers or hairpin formations, which can affect amplification specificity. Additionally, evaluating sequence similarity with other organisms ensures that the target sequences of interest do not share similarities with sequences from different organisms [12]. The technical complexity of designing a PCR system capable of distinguishing specific sequences with accuracy and reliability requires both in silico and experimental testing. Some studies have successfully distinguished NGT canola and rice varieties using PCR [11,13,14]. 

To address the challenges associated with detecting edited organisms, our approach can be applied to exploring and characterizing NGT-modified organisms, as it adheres to the principles of open science, ensuring transparency in the data and efforts for the detection, identification, and biosafety of these organisms. In this study, we present a step-by-step bioinformatics analysis to evaluate and predict primer specificity requirements and exploit, in silico and empirically, two NGT model sequences and DNA from *Arabidopsis thaliana* harboring mutation patterns with few (+1bp) or (−3 bp) nucleotides in the target growth-regulating factor (GRF) gene. Our approach may provide a theoretical foundation for assessing decisions related to accessing target specificity in future NGT organisms present in food and feed matrices supported by the current EU GMO regulations and the scientific efforts for the enforcement of the characterization of NGT modifications.

## 2. Materials and Methods

### 2.1. Overall Description of the Stepwise Approach

**Figure 1:**
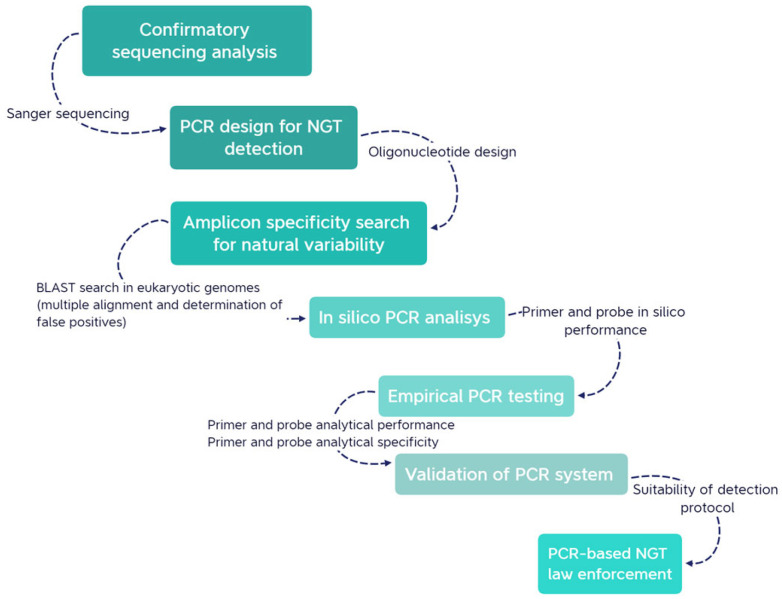
Key steps in NGT identification, detection, and quantification. The genetic analysis process begins with confirmatory sequencing analysis, primer design, and a search for amplicons to evaluate natural variability. Multi-alignment matching analysis and in silico PCR predictions need to be conducted to determine the potential for false-positive species and assess the efficiency of the chosen primer set. Empirical PCR tests focus on primer design, probe functionality, and amplification performance. To briefly evaluate specificity, both target-specific and non-target-specific amplifications may be tested to determine if the primer design can be improved. The visual guide provides a summary for methodologies for achieving the goal of detecting and quantifying small mutations in organisms resulting from NGTs.

The theoretical basis of the real-time polymerase chain reaction (RT-PCR) was used to screen mutations delivered with a CRISPR-Cas9 technique at the target site. The first step identified in our approach was the Sanger sequencing of mutated alleles for confirmatory purposes. After the confirmation of the mutation pattern in the selected genotypes, the next step was a comprehensive search of existing databases for sequence similarities and the natural variability around the mutated sequence. The outcomes of the search analysis were manually evaluated for the presence of mismatches in regions corresponding to the amplicon sequence. Lastly, an in silico PCR test was conducted to estimate the amplification rates for each PCR system, followed by a second confirmatory step to assess primer and probe specificity. The empirical phase involved the analysis of primer, probe, and amplification performance as well as an assessment of specificity. This empirical stage is crucial to ensure the functionality and reliability of the designed PCR systems. These steps are described in detail in the next sections, and a full picture of the overall approach is presented below (Figure 1). We performed the in silico step and empirical specificity primer proof in a model case for two gene-edited genotypes of *Arabidopsis thaliana*. The description of the biological material used in this study is provided in the next section.

### 2.2. Confirmatory Sequencing Analysis of CRISPR-Cas9 Mutations

The seeds from two CRISPR-Cas9 genotypes, grf1-3 and grf8-61, as well as the *Arabidopsis thaliana* control, were obtained from the Eurasian Arabidopsis Stock Center (NASC). The transformation of the *Arabidopsis thaliana* Columbia (Col-0) background was carried out using the Agrobacterium strain GV3101 through the floral dip protocol for transformation using CRISPR-Cas9 [15]. The grf1-3 genotype is a null mutant and is transgene-free. The mutation is located in the GRF1 (growth-regulating factor 1) gene, specifically in the locus AT2G22840. This mutation involves a single nucleotide polymorphism (SNP) with a guanine insertion at position 9,729,885 on the positive strand of chromosome 2. On the other hand, the grf8-61 genotype carries a three-base-pair (bp) deletion, resulting in the mutation of the GRF8 (growth-regulating factor 8) gene located at the locus AT4G24150. Additionally, the Col-0 ecotype, sequenced in the Arabidopsis Genome Initiative (https://www.ebi.ac.uk/ols/ontologies/efo/terms?short_form=EFO_0005147, access on 5 October 2022), was used as a reference genome in our study. Three individual plants per genotype were germinated in sterilized soil placed in pots. After 10 days of incubation at 4 °C in the dark, the plants were transferred to a greenhouse with a light period of 8 h/day and an intensity of approximately μmol m^−2^ s^−1^ at 25 °C for growth. Ten rosette leaves were macerated in 300 µL of Plant DNAzol^TM^ (Invitrogen^TM^, Santa Clara, CA, USA) and 10 µL of RNAse A for each sample. Subsequently, the samples were incubated in a 65 °C water bath for 30 min, followed by a 30 min ice incubation. After centrifugation at 12,000 rpm for 7 min, the supernatant was collected, and an equal volume of UltraPure phenol: chloroform: isoamyl alcohol (Invitrogen^TM^, Santa Clara, CA, USA) is added. The tubes were inverted for 3 min and then subjected to another centrifugation at the same speed and duration as previously mentioned. The resulting supernatant was collected, and an equal volume of ice-cold isopropanol was added. After ten brief tube inversions, a centrifugation step was performed. After 12 h, the precipitated DNA was centrifuged at 12,000 rpm for 7 min, and any remaining liquid was dried out. A purification process was then carried out by adding 70% ice-cold ethanol and performing new centrifugation at 13,500 rpm for 7 min. The ethanol was removed with a pipette, and the samples were air-dried. Finally, the pellet was resuspended in 50 µL of nuclease-free water. The target regions from genotypes grf1-3, grf8-61, and Columbia were amplified through conventional PCR using Sanger flankers with the following cycling conditions: 2 min at 94 °C, followed by 35 cycles of 45 s at 94 °C, 30 s at 60 °C, 1 min at 72 °C, and a final extension of 10 min at 72 °C (Applied Biosystems™ Veriti™ Thermal Cycler, Santa Clara, CA, USA). The PCR products from each genotype were purified and sequenced using the Applied Biosystems Genetic Analyzer (3500×, São Paulo, Brazil). The obtained Sanger sequences from each genotype were checked for quality using Chromas version 2.6.6 (Technelysium Pty Ltd., Brisbane, Australia). The alignment comparison with default settings (MUSCLE) was performed to compare the sequences obtained from each Sanger fragment to the GRF1 (AT2G22840; ID: 816815; Chr2) and GRF8 (AT4G24150; ID: 828515; Chr4) genes of *Arabidopsis thaliana* (NCBI GenBank taxid:3702).

### 2.3. Amplicon Sequence Search for Natural Variants

**Figure 2:**
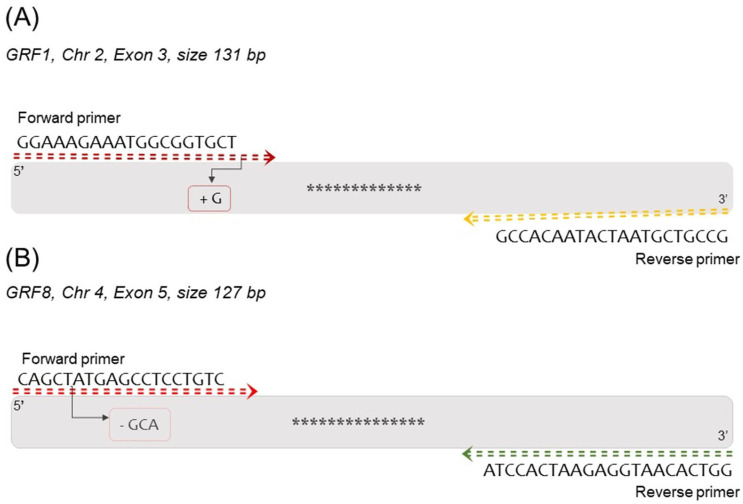
Schematic representation of the amplicon containing CRISPR-Cas9 mutations. In (**A**), the 130 bp amplicon for the grf1-3 event is illustrated, with the InDel G insert shown in the last base of the forward target primer. (**B**) displays the 127 bp amplicon from grf8-6. The asterisk represents the probe position in the amplicon. The arrow and box highlight the differences compared with the native primer sequence.

The grf1-3 and grf8-61 amplicon sequences (Table S1) and their wild-type counterpart, Columbia (Col-0), were searched against the National Center for Biotechnology Information database (NCBI) using the Basic Local Alignment Search Tool (BLAST). The databases accessed included nucleotide sequences (nt/nt) available at the GenBank + EMBL + DDBJ + PDB + RefSeq databases. Patent strings, phase strings 0, 1, and 2 HTGS, EST, STS, GSS, WGS, TSA, and greater than 100MB were excluded. To be considered as valid hits, outcome sequences retrieved through BLAST needed to have (a) an 80% coverage parameter, (b) an 80 to 98% identity level, and (c) a maximum of 10 bp of mismatches. The presence of mismatches in regions corresponding to the primer and probe sets was manually verified using the multiple sequence alignment viewer (MSA) graphic displays for NCBI nucleotide alignment. The position of the single-point mutation is indicated in Figure 2A. The positions of the exclusions indicated in Figure 2B were used to search for position polymorphisms in a database composed of 1001 variant genomes of *Arabidopsis thaliana* (https://1001genomes.org/, access on 23 October 2022). The reason behind the strategy to use amplicon instead of classical primer and probe sets was to avoid false positive hits when only one primer set or probe matched the sequence but no effective amplification was expected at similar amplicon size.

### 2.4. RT-PCR Primer and Probe Design

The design of the primers and probes for detecting CRISPR-Cas9 mutations in the grf1-3 and grf8-61 genotypes of *Arabidopsis thaliana* was carried out using the Primer-3 Plus software [16,17]. To ensure primer specificity and avoid PCR competition, the sequences of each primer set were manually verified in both DNA strand orientations to confirm the presence of the CRISPR-Cas9 mutation. First, the size of the amplicon for the single-copy reference gene served as a basis for designing the amplicons for each edited event. Subsequently, the selection of primer and probe sets was based on specific parameters. All oligonucleotides were checked for the GC content of the primers (between 45 and 55%) and the melting temperature (Tm), which was set at 3 to 5 °C below the Tm of the probe (between 62 and 68 °C) [18]. Additionally, secondary structures and hairpins were taken into consideration [12,19]. The selected primers were also assessed for their similarity to the endogenous reference gene amplicon (AT1G03400) of *Arabidopsis thaliana* [20].

### 2.5. In Silico PCR Testing 

In addition, a second confirmatory step was performed for in silico PCR testing using PrimerBLAST software [21]. This analysis aimed to screen for primer annealing in the direction from 5′ to 3′ between all combinations, including forward-to-reverse, forward-to-forward, and reverse-to-reverse. The in silico PCR testing encompassed all species available in the database, and no amplification was detected in the dataset species. For each amplicon template (grf1-3 and grf8-61), the PCR products were restricted to a size range of 100 to 300 bp, and the average melting temperature (Tm) was adjusted. The primer design details, including the positioning of the inserted base at the 3′ end of the primer, are provided in Table S2. The database analyzed consisted of eukaryotic genomes. For primer stringency, it was considered essential that the primer contained at least 2 unintended targets, with a minimum of 2 incompatibilities in the last 5 base pairs at the 3′ end. The number of target sequences retrieved for each template was considered indicative of the chances of finding amplification from different target sequences than the intended one.

### 2.6. RT-qPCR Empirical Assay 

Real-time quantitative PCR (RT-qPCR) was performed by considering Avogadro’s number, a molecular weight of 660 daltons per pair of nucleotides, and the genome size of *Arabidopsis thaliana*, which is 134,634,692 base pairs. Each PCR reaction, with a total volume of 25 μL, consisted of 20,000 copies and 5 μL of the DNA template. To optimize the primer concentration (HPLC purified, Applied Biosystems, Santa Clara, CA, USA) different combinations of forward and reverse primers were tested at three concentrations (0.200 μM, 0.400 μM, and 0.600 μM). The step-cycle program started with an initial denaturation at 95 °C for 10 min, followed by 45 cycles, each comprising 30 s at 95 °C and 1 min at 60 °C. Subsequently, nonfluorescent quencher QSY (HPLC purified, reporter FAM Applied Biosystems) probe concentrations were tested at three levels (0.100 μM, 0.200 μM, and 0.300 μM), with annealing temperatures ranging from 58 °C to 68 °C. The optimal primer and probe conditions were selected based on factors such as slope shape, cycle efficiency, fluorescence, and signal. To evaluate the specificity of the primer and probe set, the best combination was used. The step-cycle program included an initial denaturation at 95 °C for 10 min followed by 45 cycles, with each cycle consisting of 30 s at 95 °C and 1 min at 58 °C. Twelve replicates were conducted for each genotype, including two nonedited (Columbia-01 and BU-15—https://abrc.osu.edu/stocks/number/CS1035, access on 11 April 2022) and two edited (grf1-3 and grf8-61) samples. Additionally, the signal and primer compatibility for edited events was assessed, with WT Columbia tested at a concentration of 50 ng. For the single-copy reference gene (RG AT1G03400), triplicates were performed using 12.5 μL of Master Mix (Kapa Probe Force, Cape Town, South Africa), 0.400 μM forward, 0.600 μM reverse, and 0.200 μM probe. To complete the volume, IDTE was used in a 25 μL reaction volume. Negative controls were prepared in triplicate, and the assay was conducted using the Applied Biosystems™. StepOnePlus™. Santa Clara, CA, USA.

## 3. Results

### 3.1. Sequence Confirmation of Mutated Alleles

**Figure 3:**
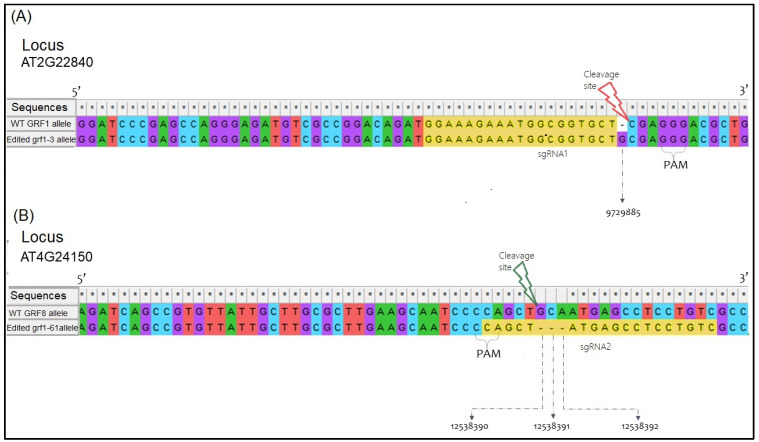
Alignment between the sequences obtained through the sequencing data from DNA strand reading 5′ to 3′; sequence represented by asterisks. A comparative analysis is presented between sequences derived from Columbia (Col-0) and the GRFs’ CRISPR-Cas9 alleles. In (**A**), the precise location of the guanine (G) insertion is indicated with an arrow in the *Arabidopsis thaliana* genome. (**B**) showcases a triplet deletion, encompassing guanine (G), cytosine (C), and adenosine (A) within the gfr8-61 CRISPR-Cas9 allele. Both genotypes exhibit mutations (insertion/deletion) localized three base pairs upstream of the Cas9 PAM cleavage. The sgRNA sequences used to develop these mutants are highlighted in yellow.

After the Sanger sequencing of the mutated genotypes and the wild-type nonmutated genotype, the intended mutations were confirmed for each new allele obtained with CRISPR-Cas9 (Figure 3). In the grf1-3 mutant, only a single addition of a guanidine was confirmed, whereas for mutant genotype grf8-61, the deletion of three base pairs starting from position 12,538,390 was observed.

### 3.2. In Silico Specificity Assessment

The search for amplicon sequences of the wild-type GRF1 gene in the NCBI nucleotide collection (nr/nt) yielded a total of 120 hits/occurrences across 39 organisms. Among these, 78 hits and 15 organisms belong to the Brassicales order (Table S3). We retrieved 58 hits associated with the genus Arabidopsis, with 56 matches attributed to *Arabidopsis thaliana* (100% identity) and the remaining 2 hits belonging to the species-genus *Arabidopsis lirata* (99.23% identity) and *Arabidopsis arenosa* (98.46% identity). In five hits, the sequences showed 100% identity as the growth-regulating protein—GRL1 gene (B2CU94_ARATH). For the wild-type GRF8 sequence, we obtained 57 hits with 100% coverage and 100% identity in *Arabidopsis thaliana* (Table S4). Of these, 25 matches were 100% identical to the *Arabidopsis thaliana* protein growth regulatory factor GRL8 gene (B2CUI8_ARATH). These results indicate that no significant homology exists for the two gene alleles outside the Arabidopsis genus (Figure S2).

For the grf1-3 CRISPR-Cas9 amplicon (mutated), we identified 36 hits with sequences showing 100% coverage. In addition to the two Arabidopsis species that displayed full coverage at 100%, namely *Arabidopsis lirata* and *Arabidopsis arenosa*, these two species exhibited identical sequences, with an SNP variation at position 15. Furthermore, *A. arenosa* exhibited a mismatch at position 108. Other species, such as *Camelina sativa* and *Camelina hispida*, showed 100% coverage but lower identity levels, with values of 98.48% and 97.71%, respectively, due to different mismatches. We also encountered 10 hits from 4 species within the Brassica genus, demonstrating 95.5% identity. *Raphanus sativus* presented two sequence hits, with coverage and identity levels of 98% and 96.12%, respectively. No other polymorphisms were detected in the *Arabidopsis thaliana* database for the provided amplicons. The matching results for grf1-3 are presented in Table 1.

For the grf8-1 amplicon, 26 hits were recovered with 96% coverage due to the presence of gaps in the initial bases, corresponding to (C, A, G, C, T) of the forward primer. However, after the 6-mer position, the sequences exhibited 100% coverage. Among these hits, two displayed an identity of 97.54% due to mismatches in the probe sequence. Additionally, two other hits showed a 99.20% identity and 98% coverage, with a mismatch in the 5′ 5-mer, representing a transversion-type mutation at position 12,538,392 in the forward primer. No other sequences were found with 100% alignment for this amplicon. The variation observed in these hits suggests that this 5 pb region may consist of polymorphic nucleotides. Nevertheless, the three excluded bases were not located in the variant database for *Arabidopsis thaliana.* Table 2 compiles the results obtained for the grf8-61 amplicon search against the database.

### 3.3. In Silico PCR Performance

A pair of primers should ideally amplify only the target sequence. However, this can be particularly challenging when the target region differs by one or a few nucleotides from other potential targets. The in silico PCR results revealed 57 potential hits for the grf1-3 PCR system. Among these, 39 hits were associated with the forward primer, which contained a cytosine (C) base inserted as a mismatch. The remaining 18 hits were distributed in *Arabidopsis hispida* (1 hit), *Arabidopsis arenosa* (1), and *Brassica* spp. (16), although these hits contained mismatches in the amplicon against *Brassica napus*, *Brassica oleracea,* and *Brassica* spp. (Table 3; Figure S1). These findings suggest that the primer set designed to distinguish the grf1-3 CRISPR-Cas9 genotype may have the potential to amplify sequences in other species, such as Arabidopsis and Brassica.

The primer set designed for detecting the 3 bp deletion in the growth-regulating factor 8 gene exhibited a single unique target within *Arabidopsis thaliana* (Table 4). Hence, it can be considered specific for the grf8-61 genotype, as indicated by the in silico PCR prediction.

### 3.4. Real-Time qPCR Performance and Empirical Primer Specificity Evaluation

**Figure 4:**
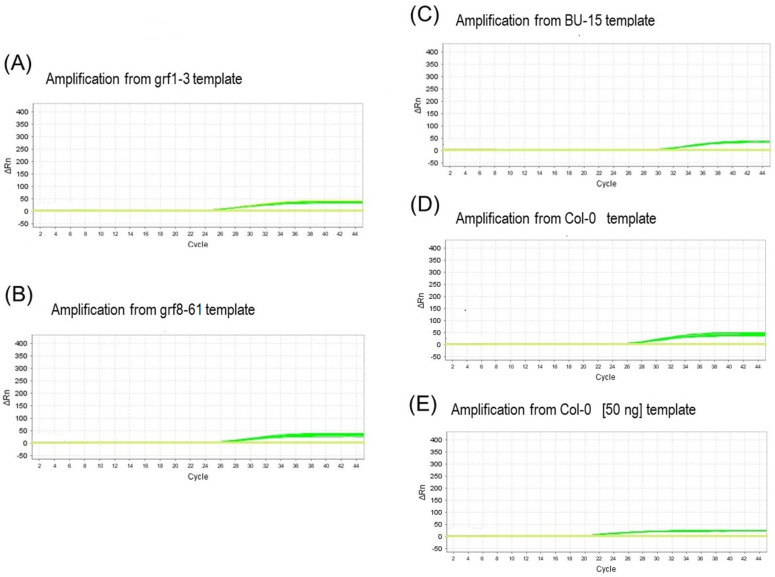
Specificity assay. Four genotypes (**A**–**D**), each with 20,000 DNA copies, were subjected to identical RT-qPCR conditions using the primer grf1-3. Notably, all four genotypes exhibited similar amplification profiles. For the negative control (**E**), Columbia-0, when a higher amount of DNA was used (50 ng, 17 times), amplification occurred earlier.

The genotype (template) grf1-3 consistently produced similar Ct values across the nine different conditions using the grf1-3 primer (maximum of 21.61, average of 20.96, and minimum of 20.48). The condition with 0.400 μM for both forward and reverse primers appeared to be the most optimal, generating a Ct of 20.77. The amplification curve exhibited a characteristic plateau with a very low delta RN. Three samples display a noise spike flag in Table S5. The probe performance and annealing temperature showed that using 0.200 μM of the probe at 58 °C resulted in a more efficient curve (Ct of 23.22). This condition also achieved fluorescence amplification (delta RN 50) at 58 °C when an automatic threshold was applied. The single-copy primer RG successfully amplified both the samples with the edited event and the control samples. For the nine combinations of the grf8-61 template and grf8-61 primer, the Ct values varied between a maximum of 25.08, an average of 24.99, and a minimum of 24.20, and all samples exhibited a noise spike flag and very low fluorescence. Nevertheless, the condition using 0.400 μM for forward and 0.200 μM for reverse was selected for the subsequent probe and temperature annealing assays. The primer’s low efficiency was confirmed in this final performance, demonstrating minimal amplification at all tested temperatures. Consequently, this primer was excluded from further analysis, provoking a consideration of alternative strategies and investments to enhance the target specificity. To assess the proof of concept regarding the specificity of the grf1-3 primer, we conducted an assay involving different genotypes (DNA templates) with 12 replicates. The results revealed that the grf1-3 primer lacked specificity in distinguishing between genotypes edited by CRISPR-Cas9 (Figure 4A,B). Both the grf1-3 template (Ct 23.99) and the grf8-61 genotype (Ct 25.02), under the same conditions of oligonucleotide concentration, cycling parameters, and DNA content (20.00 copies), exhibited amplification. This similarity was also observed in two other negative control genotypes (Figure 4C,D). For the BU-15 template, higher Ct values (29.36) were observed compared with the Ct values (25.25) of the Col-0 genotype. Notably, when DNA from the Col-0 control genotype was used in high concentrations (50 ng), it resulted in the expected amplification (average Ct of 18.91) for a nonmutant genotype. This highlights that the presence of the mutated base inserted in the 20-mer did not confer specificity in distinguishing between genotypes of the *Arabidopsis thaliana* species. To test the hypothesis of distinguishing between these genotypes using this set of primers, it will be necessary to enhance the specificity, potentially through chemical blocking of the nitrogenous base.

## 4. Discussion 

From simple genetic modification and traditional genetic modification methods to the more recent genome editing methods, restriction enzymes or nucleases have been employed to create breaks in DNA double strands (DBSs) that are repaired through two major pathways. One pathway involves joining the ends using a template (HR), while the other pathway joins the ends without homology (NHEJ). In plants produced through new breeding techniques (NBTs), the NHEJ pathway is predominantly used to achieve gene knockout and create mutants with desired agricultural traits [22,23]

Mutants generated with this repair mechanism exhibit one or a few insertion/deletion (InDel) mutations in addition to their unmutated parts, and these distinct nucleotides serve as identifiers to characterize the new genetically modified organism (GMO). In this study, two regions of transcriptional growth regulatory genes (GRFs) in *Arabidopsis thaliana* were chosen as case studies to develop a stepwise approach for the in silico prediction of specific PCR primer sets. The GRF protein gene family is involved in growth and development as well as the stress response [24,25].

Our proof of concept for specificity introduced known concepts from GMO method guidelines linked to EFSA. It involved exercises in both theoretically predicting and empirically testing qPCR, amplicon, and primer set specificity, taking into consideration single-nucleotide insertions and three-base deletions. The first phase of this approach involved an in silico screening of amplicon sequences from the native genes of *A. thaliana* Columbia-0, sourced from the NCBI nucleotide database. This screening allowed us to identify that the primer sequence used in the GRF1 gene has a 99% identity with *Arabidopsis lyrate*; 97.24% with *Arabidopsis arenosa;* and 94.6% with agricultural species such as *Brassica napus, Brassica rapa, Brassica rapa subsp. Pekinensis,* and *Brassica oleracea var oleracea*. Additionally, we found 15 other organisms belonging to the order Brassicales with lower similarity, having more than six base-pair mismatches. The amplicon sequence search for the GRF8 gene showed only one match in *Arabidopsis thaliana* with 100% identity, confirming the native sequence.

As expected, the BLAST analysis of amplicon grf1-3 indicated that the single nucleotide polymorphism was the only difference in 35 sequence hits with 99.8% similarity. Similar results were found by [13] when analyzing amplicons of primer sets for the detection of gene-edited canola. In their study, the BLAST search produced hits in *B. oleracea* and *B. carinata*, where the only difference between the targets was the last nucleotide at position 22. In this position, the complementary reverse primer was located for detection [13].

Most gene-edited organisms contain mutations in conserved regions and relevant exons required for achieving specific traits. This is the case for gene-edited *Camelina sativa* and *Brassica napus*, created using CRISPR-Cas9 to increase the oleic acid content [26,27,28]. This is also observed in the fungus *Verticillium longisporum*-resistant *Arabidopsis thaliana* and *Brassica napus*, which contain a unique base transition in the CRT1a gene (calreticulin) [29]. While grf1-3 and grf8-61 genotypes are exclusive variants, meaning they do not have polymorphic positions found in the *Arabidopsis thaliana* 1001 variants database, the results of the in silico PCR were distinctive for each genotype. The primer set designed for the genotype grf1-3 does not distinguish the mutant event from 39 other sequence hits observed in *Arabidopsis arenosa*, *Arabidopsis lyrata*, *Camelina sativa,* and *Brassica spp*. This means that, for example, in food mixtures containing *Arabidopsis thaliana* and Camelina sativa, the primer set may not be able to differentiate the modified CRISPR-Cas9 event for the grf1-3 genotype. For the grf8-61 genotype, the results show that the primer set was specific for detecting the new genotype produced with CRISPR-Cas9, as confirmed by the NCBI genetic database search.

RT-qPCR, a method based on hydrolysis probe chemistry, is the most widely used method for GMO detection in Europe [9]. From a detection perspective, these GMOs must exhibit unique amplification products for which the method was designed [8] Additionally, they should differentiate their products from naturally occurring variants and those obtained through conventional mutagenesis [30]. Although real-time PCR amplification is influenced by various factors such as primer size, SNP position, hybridization, Taq polymerase fidelity, and other PCR conditions, it is critical to achieve stable, nondisruptive thermodynamic energy at the 3′ end of the primer sequences to enhance specificity [31,32]. Previous studies have demonstrated the challenge of differentiating genotypes that have only a one-base difference. Our results, despite using the efficiency of a hot-start Taq polymerase, demonstrate the nonadherence of the end that has the difference with the wild-type sequence (Figure 4).

For NGT products, the absence of inserted foreign DNA sequences is a distinguishing feature. The event-specific identification of NGT plant products, rather than just the detection of the InDel/single nucleotide variant (SNV), appears to pose a considerably greater challenge, particularly when the detected genome edit needs to be distinguished from conventional plant products with identical sequences. The question arises as to whether the detection of a characteristic SNV or InDel at a specific site in the genome of the NGT plant is sufficient for its identification according to EU legislation [33]. Previous studies have used amplicon and primer sets to determine whether a single-point variation in a gene, involving a single nucleotide (adenosine) insertion in the rice variety OsMADS26 (locus: Os08g02070), could be distinguished. The specificity of the method in distinguishing the modification introduced by gene editing technology through its single variation point was achieved using a 2-plex digital droplet PCR method [11]. Similarly, in rice, the identification of genotypes with InDels ranging from 1 to 18 base pairs (bps) for the chlorophyll oxygenase 1 (CAO1) gene region was empirically demonstrated through real-time quantitative (qPCR) and digital droplet (ddPCR) PCR. In both PCR systems, it was possible to estimate the genome-edited ingredient content without relying on an in silico approach [14].

In our study, we investigated the specificity of two different primer sets using the in silico prediction of potential sequence targets in the entire public database available at NCBI. In addition, we performed empirical testing of these primer sets in order to evaluate their analytical specificity across different genotypes. For the grf1-3 primer set, our in silico results were supported by our empirical analysis, demonstrating a lack of analytical specificity for the grf1-3 primer in other *Arabidopsis thaliana* genotypes even in the presence of nucleotide mismatches. Surprisingly, the grf8-61 primer indicated a high in silico discriminatory capacity, See Supplementary Materials Figure S1 but empirical amplification was not consistent under the conditions tested, and the primer is not considered efficient for the grf8-61 event amplification (See Supplementary Materials Table S5).

In a prior study by Chhalliyil [34] and co-authors (2022), empirical data showed the specificity of a primer set based on a locked nucleic acids (LNAs) strategy for detecting and identifying the first commercialized genome-edited plant, Cibus canola, containing two mutated genes, AHAS1C and AHAS3A. Later that same year, Weidner [13] and co-authors (2022) showed that the method might not be specific to the GMO event, but could also result in spurious amplification in other Brassica species. Therefore, wet lab laboratory testing will have to consider chemical modifications in the PCR system to increase specificity, such as locked nucleic acid technology (LNA), RNAse-H systems, etc. [35], and/or ddPCR strategies to provide specificity in the detection of mutant and wild-type variants simultaneously [11].

RT-qPCR, widely used for GMO detection in Europe, requires the development of unique amplification products. While several factors influence real-time PCR amplification, such as primer size, SNP position, hybridization, Taq polymerase fidelity, and other PCR conditions, enhancing specificity through stable thermodynamic energy at the 3′ end of primer sequences remains a critical consideration. Empirical PCR testing represents the ultimate proof of method specificity, but an in silico prediction can anticipate the failure to distinguish amplicons from different organisms. Therefore, our study provides a stepwise approach to the search for amplicon and primer set specificity in available databases. This approach paves the way for defining minimum quality performance criteria in GE plant detection, which is essential for food safety management and the global food trade [36]. Additionally, it allows the development of strategies to increase the amplification specificity using targeted high-throughput sequencing for detecting specific single nucleotide variants (SNVs) in CRISPR-Cas9 genome-edited plants. Although this approach shows great promise, its current implementation in GMO control is challenging. Future studies should aim to accumulate sufficient data for further performance assessments and address technical and analytical challenges, including the complexities of plant genomes and food/feed products. Moreover, the commercialization of a genome-edited organism in the European market would require a full validation process for the proposed sequencing approach, including assessments of transferability and robustness, before adoption by enforcement laboratories [37]. While empirical PCR testing represents the ultimate proof of method specificity, our stepwise approach provides a valuable tool for evaluating and ensuring minimum quality performance criteria for specificity primers in GMO detection. Additionally, given that genome-edited plants are expected to become increasingly prevalent in food and feed matrices, *Arabidopsis thaliana*, as a well-sequenced genomic plant model with a broad degree of knowledge about natural variants, serves as an excellent case study. 

## 5. Conclusions 

In this paper, we used *Arabidopsis thaliana* mutants as a model plant to demonstrate the applicability of our in silico methodology in a case study for the prediction of primer specificity via a public genomic databases search. The findings indicate that a primer set designed for the grf1-3 genotype, which contains a single nucleotide polymorphism, may potentially generate amplicons in other species. The in silico prediction showed that the primer set failed to effectively distinguish between the grf1-3 genotype and other species within the same genus (i.e., *A. lirata* and other phylogenetically related species of *C. Sativa* and *Brassica* spp.). Empirical results confirmed that the primer set designed for the grf1-3 genotype indeed produces amplicons in other Arabidopsis species. On the other hand, the in silico prediction analysis for the grf8-61 primer set, our second mutant genotype, showed an effective discrimination of this event from other organism sequences in databases. Our study shows the importance of considering database results in conjunction with the performance of the primers/probe PCR systems as a critical step when planning PCR-based methods for the detection, identification, and quantification of GMO events in light of EU regulations and law enforcement. The implication regarding the feasibility of detection is a significant effort aimed at facilitating the commercialization and safety assessment of genetically modified organisms (GMOs). This endeavor has the potential to be highly valuable, especially in the context of labeling NTG organisms. 

## Figures and Tables

**Table 1:** The BLAST hits retrieved from the *grf1-3* CRISPR-Cas9 amplicon sequence.

Sequence Number	Hits	Accession	BLAST against the Query grf1-3		(MSA) Mismatches (bp) or Gaps against Each Accession		
			Query Cover	Per. Ident	Forward	Probe	Reverse
1	*Arabidopsis thaliana* genome assembly, chromosome: 2	LR782543.1	100%	99.24%	1	0	0
2	*Arabidopsis thaliana* genome assembly, chromosome: 2	LR699746.2	100%	99.24%	1	0	0
3	*Arabidopsis thaliana* genome assembly, chromosome: 2	LR699771.1	100%	99.24%	1	0	0
4	*Arabidopsis thaliana* genome assembly, chromosome: 2	LR699766.1	100%	99.24%	1	0	0
5	*Arabidopsis thaliana* genome assembly, chromosome: 2	LR699761.1	100%	99.24%	1	0	0
6	*Arabidopsis thaliana* genome assembly, chromosome: 2	LR699756.1	100%	99.24%	1	0	0
7	*Arabidopsis thaliana* genome assembly, chromosome: 2	LR699751.1	100%	99.24%	1	0	0
8	*Arabidopsis thaliana* genome assembly, chromosome: 2	LR215053.1	100%	99.24%	1	0	0
9	*Arabidopsis thaliana* growth-regulating factor 1 (GRF1), mRNA	NM_127849.4	100%	99.24%	1	0	0
10	*Arabidopsis thaliana* chromosome 2	CP116281.1	100%	99.24%	1	0	0
11	*Arabidopsis thaliana* genome assembly, chromosome: 2	OX298798.1	100%	99.24%	1	0	0
12	*Arabidopsis thaliana* genome assembly, chromosome: 2	OX298803.1	100%	99.24%	1	0	0
13	*Arabidopsis thaliana* ecotype 1254 chromosome 2 sequence	CP086755.1	100%	99.24%	1	0	0
14	*Arabidopsis thaliana* ecotype 5856 chromosome 2 sequence	CP086750.1	100%	99.24%	1	0	0
15	*Arabidopsis thaliana* ecotype 6021 chromosome 2 sequence	CP086745.1	100%	99.24%	1	0	0
16	*Arabidopsis thaliana* ecotype 6024 chromosome 2 sequence	CP086740.1	100%	99.24%	1	0	0
17	*Arabidopsis thaliana* ecotype 9412 chromosome 2 sequence	CP086735.1	100%	99.24%	1	0	0
18	*Arabidopsis thaliana* ecotype 9470 chromosome 2 sequence	CP086730.1	100%	99.24%	1	0	0
19	*Arabidopsis thaliana* chromosome 2	CP087127.2	100%	99.24%	1	0	0
20	*Arabidopsis thaliana* isolate t2t_salk_col chromosome 2	CP096025.1	100%	99.24%	1	0	0
21	*Arabidopsis thaliana* genome assembly, chromosome: 2	OW119597.1	100%	99.24%	1	0	0
22	*Arabidopsis thaliana* genome assembly, chromosome: 2	LR881467.1	100%	99.24%	1	0	0
23	*Arabidopsis thaliana* genome assembly, chromosome: 2	LR797808.1	100%	99.24%	1	0	0
24	*Arabidopsis thaliana* genome assembly, chromosome: 2	LR797803.1	100%	99.24%	1	0	0
25	*Arabidopsis thaliana* genome assembly, chromosome: 2	LR797798.1	100%	99.24%	1	0	0
26	*Arabidopsis thaliana* genome assembly, chromosome: 2	LR797793.1	100%	99.24%	1	0	0
27	*Arabidopsis thaliana* genome assembly, chromosome: 2	LR797788.1	100%	99.24%	1	0	0
28	*Arabidopsis thaliana* chromosome 2	CP002685.1	100%	99.24%	1	0	0
29	*Arabidopsis thaliana* At2g22840 mRNA for hypothetical protein, partial cds, clone: RAAt2g22840	AB493560.1	100%	99.24%	1	0	0
30	*Arabidopsis thaliana* isolate CS906 GRL1 (GRL1) gene, partial cds	EU550462.1	100%	99.24%	1	0	0
31	*Arabidopsis thaliana* isolate CS902 GRL1 (GRL1) gene, partial cds	EU550456.1	100%	99.24%	1	0	0
32	*Arabidopsis thaliana* isolate CS6799 GRL1 (GRL1) gene, partial cds	EU550455.1	100%	99.24%	1	0	0
33	*Arabidopsis thaliana* isolate CS901 GRL1 (GRL1) gene, partial cds	EU550445.1	100%	99.24%	1	0	0
34	*Arabidopsis thaliana* transcription activator (GRF1) mRNA, complete cds	AY102634.1	100%	99.24%	1	0	0
35	*Arabidopsis thaliana* chromosome 2 clone T20K9 map CIC06C07, complete sequence	AC004786.3	100%	99.24%	1	0	0
36	*Arabidopsis thaliana* Full-length cDNA Complete sequence from clone GSLTPGH12ZD08 of Hormone-Treated Callus of strain col-0 of *Arabidopsis thaliana (thale cress)*	BX820248.1	100%	99.24%	1	0	0
37	PREDICTED: *Arabidopsis xampl* subsp. xampl growth-regulating factor 1 (LOC9316532), mRNA	XM_002878592.2	100%	98.47%	1	0	0
38	PREDICTED: *Camelina sativa* growth-regulating factor 1-like (LOC104713726), mRNA	XM_010430916.2	100%	98.47%	1	0	0
39	PREDICTED: *Camelina sativa* growth-regulating factor 1 (LOC104751923), mRNA	XM_010473979.2	100%	98.47%	1	0	0
40	PREDICTED: *Camelina sativa* growth-regulating factor 1-like (LOC104704976), mRNA	XM_010420970.1	100%	98.47%	1	0	0
41	*Camelina hispida* cultivar hispida voucher DAO 902780 chromosome 2	CP094632.1	100%	97.71%	1	0	1
42	*Arabidopsis arenosa* genome assembly, chromosome: 4	LR999454.1	100%	97.71%	1	0	1
43	*Raphanus sativus* genome assembly, chromosome: 6	LR778315.1	98%	96.12%	1	0	2 (gap)
44	PREDICTED: *Raphanus sativus* growth-regulating factor 1 (LOC108836427), mRNA	XM_018609585.1	98%	96.12%	1	0	2 (gap)
45	PREDICTED: *Brassica rapa* growth-regulating factor 1 (LOC103858395), mRNA	XM_009135745.3	100%	95.42%	1	0	1
46	*Brassica oleracea* HDEM genome, scaffold: C3	LR031872.1	100%	95.42%	1	0	1
47	*Brassica rapa* genome, scaffold: A03	LR031572.1	100%	95.42%	1	0	1
48	PREDICTED: *Capsella rubella* growth-regulating factor 1 (LOC17887921), mRNA	XM_006293922.2	100%	95.42%	1	1	1
49	PREDICTED: *Brassica napus* growth-regulating factor 1-like (LOC125584397), mRNA	XM_048752816.1	100%	95.42%	1	0	1
50	PREDICTED: *Brassica napus* growth-regulating factor 1 (LOC106389497), mRNA	XM_013829762.3	100%	95.42%	1	0	1
51	*Brassica rapa* genome assembly, chromosome: A03	LS974619.2	100%	95.42%	1	0	1
52	*Brassica napus* genome assembly, chromosome: C03	HG994367.1	100%	95.42%	1	0	1
53	*Brassica napus* genome assembly, chromosome: A03	HG994357.1	100%	95.42%	1	0	1
54	*Brassica rapa subsp.* Pekinensis growth-regulating xamp 1 mRNA, partial cds	JN698986.1	100%	95.42%	1	0	1
55	PREDICTED: *Brassica oleracea var. oleracea* growth-regulating factor 1 (LOC106328366), mRNA	XM_013766798.1	100%	94.66%	1	0	1
56	PREDICTED: *Eutrema salsugineum* growth-regulating factor 1 (LOC18021800), mRNA	XM_006404687.2	100%	93.89%	1	2	2
57	*Arabis alpina* genome assembly, chromosome: 6	LT669793.1	93%	95.90%	1	2	9 (gap)

**Table 2:** The BLAST hits retrieved from the *grf8-61* CRISPR-Cas9 amplicon sequence.

Sequence Number	Description	Accession	BLAST against the Query grf8-61		(MSA) Mismatches (bp) or Gaps against Each Accession		
			Query Cover	Per. Ident	Forward	Probe	Reverse
1	*Arabidopsis thaliana* genome assembly, chromosome: 4	LR782545.1	96%	100.00%	0	0	5 (gaps)
2	*Arabidopsis thaliana* genome assembly, chromosome: 4	LR699748.2	96%	100.00%	0	0	5 (gaps)
3	*Arabidopsis thaliana* genome assembly, chromosome: 4	LR699773.1	96%	100.00%	0	0	5 (gaps)
4	*Arabidopsis thaliana* genome assembly, chromosome: 4	LR699768.1	96%	100.00%	0	0	5 (gaps)
5	*Arabidopsis thaliana* genome assembly, chromosome: 4	LR699758.1	96%	100.00%	0	0	5 (gaps)
6	*Arabidopsis thaliana* genome assembly, chromosome: 4	LR699753.1	96%	100.00%	0	0	5 (gaps)
7	*Arabidopsis thaliana* genome assembly, chromosome: 4	LR215055.1	96%	100.00%	0	0	5 (gaps)
8	*Arabidopsis thaliana* chromosome 4	CP116283.1	96%	100.00%	0	0	5 (gaps)
9	*Arabidopsis thaliana* genome assembly, chromosome: 4	OX298800.1	96%	100.00%	0	0	5 (gaps)
10	*Arabidopsis thaliana* genome assembly, chromosome: 4	OX298805.1	96%	100.00%	0	0	5 (gaps)
11	*Arabidopsis thaliana* ecotype 1254 chromosome 4 sequence	CP086757.1	98%	99.20%	0	0	4 (gaps) 1 mismatch
12	*Arabidopsis thaliana* ecotype 5856 chromosome 4 sequence	CP086752.1	96%	100.00%	0	0	5 (gaps)
13	*Arabidopsis thaliana* ecotype 6021 chromosome 4 sequence	CP086747.1	96%	100.00%	0	0	5 (gaps)
14	*Arabidopsis thaliana* ecotype 6024 chromosome 4 sequence	CP086742.1	96%	100.00%	0	0	5 (gaps)
15	*Arabidopsis thaliana* ecotype 9412 chromosome 4 sequence	CP086737.1	96%	100.00%	0	0	5 (gaps)
16	*Arabidopsis thaliana* ecotype 9470 chromosome 4 sequence	CP086732.1	96%	100.00%	0	0	5 (gaps)
17	*Arabidopsis thaliana* chromosome 4	CP087129.2	96%	100.00%	0	0	5 (gaps)
18	*Arabidopsis thaliana* isolate t2t_salk_col chromosome 4	CP096027.1	96%	100.00%	0	0	5 (gaps)
19	*Arabidopsis thaliana* genome assembly, chromosome: 4	OW119599.1	96%	100.00%	0	0	5 (gaps)
20	*Arabidopsis thaliana* genome assembly, chromosome: 4	LR881469.1	96%	100.00%	0	0	5 (gaps)
21	*Arabidopsis thaliana* genome assembly, chromosome: 4	LR797810.1	96%	100.00%	0	0	5 (gaps)
22	*Arabidopsis thaliana* genome assembly, chromosome: 4	LR797805.1	96%	100.00%	0	0	5 (gaps)
23	*Arabidopsis thaliana* genome assembly, chromosome: 4	LR797800.1	96%	100.00%	0	0	5 (gaps)
24	*Arabidopsis thaliana* genome assembly, chromosome: 4	LR797795.1	96%	100.00%	0	0	5 (gaps)
25	*Arabidopsis thaliana* chromosome 4	CP002687.1	96%	100.00%	0	0	5 (gaps)
26	*Arabidopsis thaliana* DNA chromosome 4, contig xample No. 61	AL161561.2	96%	100.00%	0	0	5 (gaps)
27	*Arabidopsis thaliana* DNA chromosome 4, BAC clone T19F6, partial sequence (ESSA xample)	AL109619.1	96%	100.00%	0	0	5 (gaps)
28	*Arabidopsis thaliana* chromosome IV BAC T19F6 genomic sequence, complete sequence	AC002343.1	98%	99.20%	0	0	4 (gaps) 1 mismatch
29	*Arabidopsis thaliana* genome assembly, chromosome: 4	LR699763.1	96%	97.54%	0	2	5 (gaps)
30	*Arabidopsis thaliana* genome assembly, chromosome: 4	LR797790.1	96%	97.54%	0	2	5 (gaps)
31	*Arabidopsis thaliana* growth-regulating factor 8 (GRF8), partial mRNA	NM_118547.2	83%	100.00%			

**Table 3:** Summary of in silico PCR amplification for the grf1-3 amplicon.

Total of Mismatches	Number/Hits Analyzed	Sequences Corresponding Perfectly to the Primer	Number of BLAST Hits Recovered	Possible Discrimination between the grf1-3 Genotype and Other Lines
1	39	0	57	Low
2	17	0		
3	1	0		

**Table 4:** Summary of in silico PCR amplification for the grf8-61.

Total of Mismatches	Number/Hits Analyzed	Sequences Corresponding Perfectly to the Primers	Number of BLAST Hits Recovered	Possible Discrimination between the grf8-3 Genotype and Other Lines
1	1	1	1	High

## Data Availability

The data used to support the findings of this study can be made available by the corresponding author upon request.

## Supplementary Materials

The following supporting information can be downloaded at: https://www.mdpi.com/article/10.3390/foods12234298/s1, Table S1: CRISPR-Cas9 amplicon sequences; Table S2: Oligonucleotides parameters; Table S3: Results from the GRF1 gene ecotype Columbia (*A. thaliana*) sequence searches against the forward reverse and probe sequences in the NCBI (nucleotide database); Table S4: Results from the GRF8 gene ecotype Columbia (*A. thaliana*) sequence searches against the forward reverse and probe sequences in the NCBI (nucleotide database); Table S5: Primer grf8-61 with grf8-61 template; Figure S1: Confirmatory in silico specificity primer analysis for each allele in Arabidopsis genus NCBI database; Figure S2: Results from in silico PCR. The Panel A shows the summarized result for the grf1-3 amplicon as template. Panel B, shows the grf8-61 amplicon.
