# Supplementary material for: Specificity Testing for NGT PCR-Based Detection Methods in the Context of the EU GMO Regulations
Source: Foods. 2023 Nov 28;12(23):4298. doi: 10.3390/foods12234298 (PMC10706100; doi:10.3390/foods12234298)
Supplement: Supplementary file 1 [file foods-12-04298-s001.zip › Figure S1.pptx]

## Slide 1
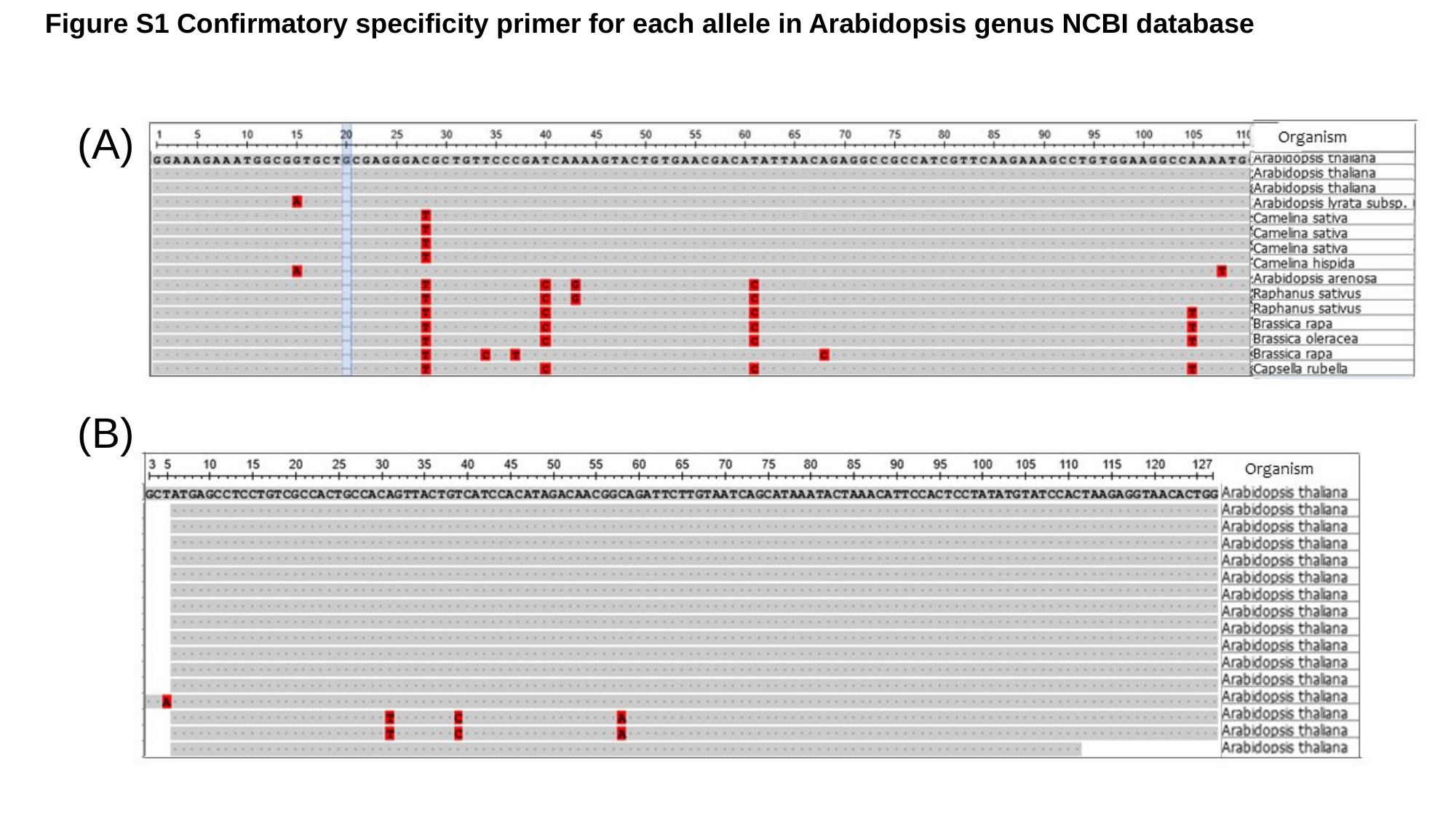

Figure S1 Confirmatory specificity primer for each allele in Arabidopsis genus NCBI database
(A)
(B)
