# Supplementary material for: Specificity Testing for NGT PCR-Based Detection Methods in the Context of the EU GMO Regulations
Source: Foods. 2023 Nov 28;12(23):4298. doi: 10.3390/foods12234298 (PMC10706100; doi:10.3390/foods12234298)
Supplement: Supplementary file 1 [file foods-12-04298-s001.zip › Figure S2.pptx]

## Slide 1
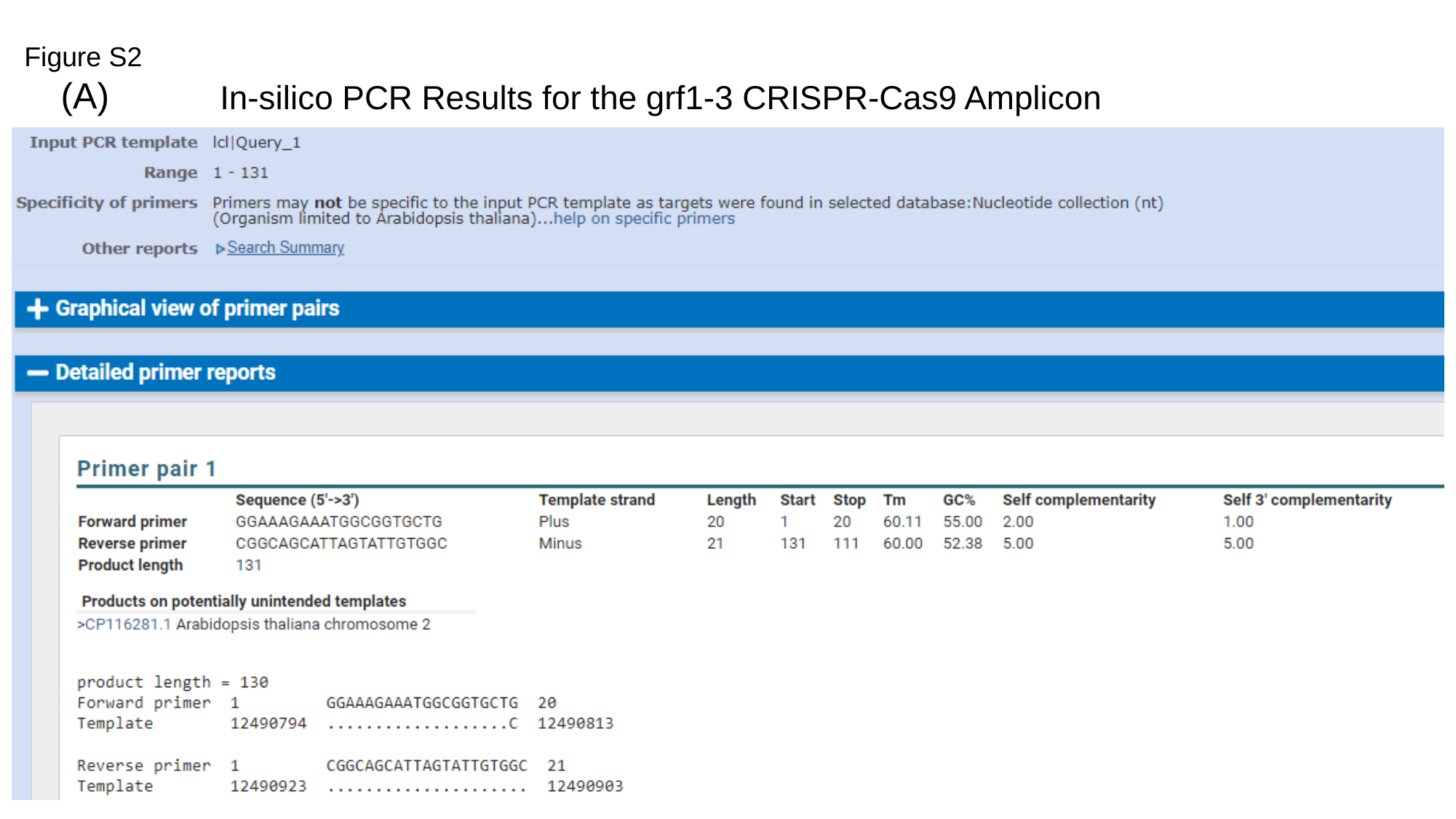

Figure S2
(A)
In-silico PCR Results for the grf1-3 CRISPR-Cas9 Amplicon

## Slide 2
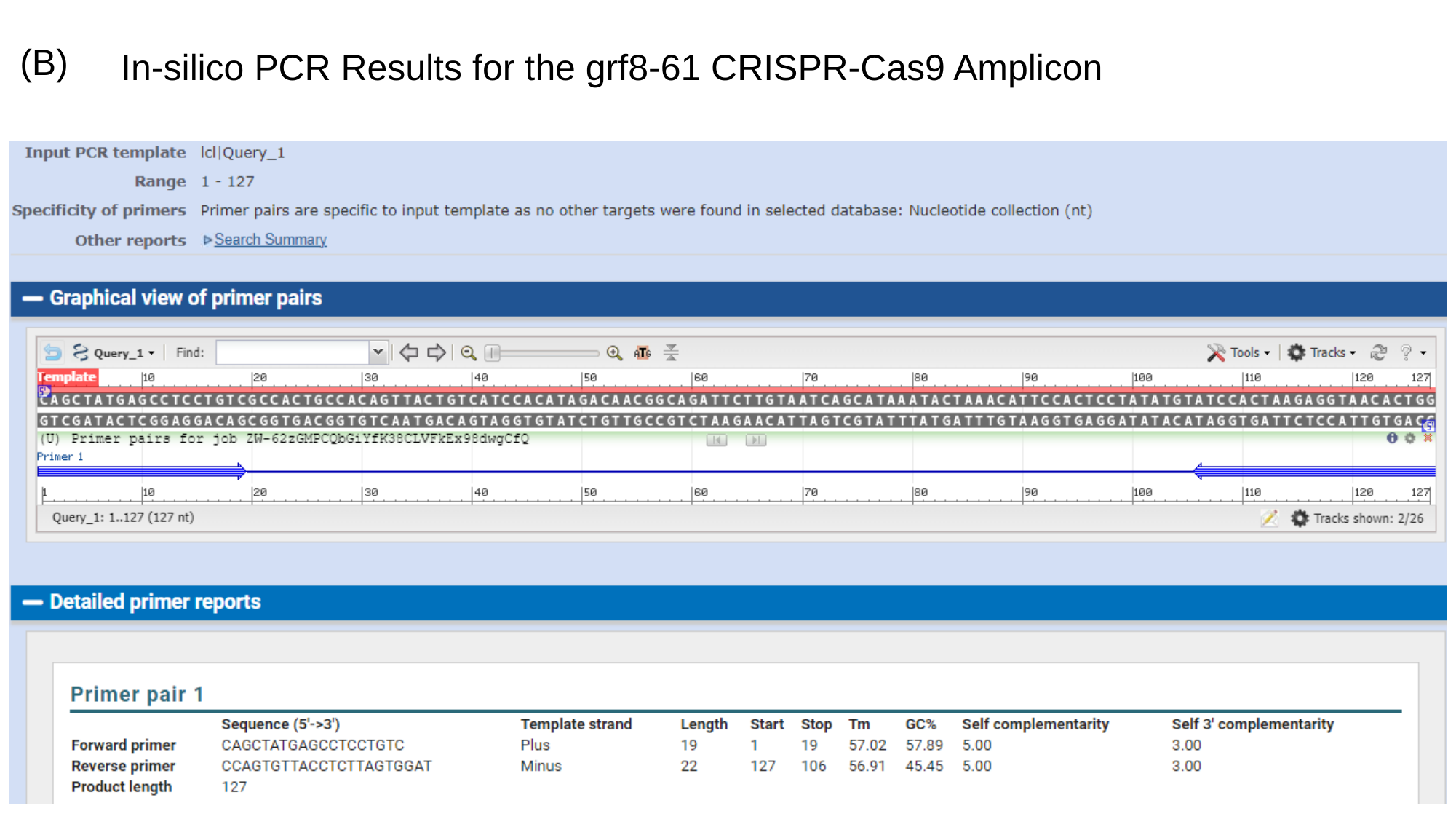

(B)
In-silico PCR Results for the grf8-61 CRISPR-Cas9 Amplicon
