# Supplementary material for: Specificity Testing for NGT PCR-Based Detection Methods in the Context of the EU GMO Regulations
Source: Foods. 2023 Nov 28;12(23):4298. doi: 10.3390/foods12234298 (PMC10706100; doi:10.3390/foods12234298)
Supplement: Supplementary file 1 [file foods-12-04298-s001.zip › Table S1 .pdf]

Supplementary Materials

Table S1 CRISPR-Cas9 amplicon sequences

grf1-3 Sequence 5'3'
GGAAAGAAATGGCGGTGCTGCGAGGGACGCTGTTCCCGATCAAAAGTACTGTGAACGACATATTAACAGAGGCCGCCATCGTTCAAGAAAGCCTGTGGAAGGCCAAAATGGCCACAATACTAATGCTGCCG

grf8-64 Sequence 5'3'
CAGCTATGAGCCTCCTGTCGCCACTGCCACAGTTACTGTCATCCACATAGACAACGGCAGATTCTTGTAATCAGCATAAATACTAAACATTCCACTCCTATATGTATCCACTAAGAGGTAACACTGG
