# Supplementary material for: Specificity Testing for NGT PCR-Based Detection Methods in the Context of the EU GMO Regulations
Source: Foods. 2023 Nov 28;12(23):4298. doi: 10.3390/foods12234298 (PMC10706100; doi:10.3390/foods12234298)
Supplement: Supplementary file 1 [file foods-12-04298-s001.zip › Table S2.pdf]

Table S2 Oligonucleotides information

Name	Sequence 5'3'	Expected MT	GC	Amplicon size
grf1_3 Forward	GGAAAGAAATGGCGGTGCTG	63.0	55.0	
grf1_3 Reverse	CGGCAGCATTAGTATTGTGGC	63.0	52.4	131 bp
Probe	ACAGAGGCCGCCATCGTTCA			
grf8_61 Forward	CAGCTaATGAGCCTCCTGTC	62	45.5	127 bp
grf8_61 Reverse	CCAGTGTTACCTCTTAGTGGAT	63	57.9	
Probe	CTGCCGTTGTCTATGTGGATGACAGT			
AT1G03400 Forward	GCGGAGCATAGGGTGATAGC			
AT1G03400 Reverse	TGTAACTTAGGAGCATCGAGCG	Yang et al., 2018		209 bp
Probe	ATGGGCCAATCAAAGATCTCCTGTCTGC			

Target modification
AT1G03400 is the endogenous gene used as a length parameter for amplicons
