# Supplementary material for: Specificity Testing for NGT PCR-Based Detection Methods in the Context of the EU GMO Regulations
Source: Foods. 2023 Nov 28;12(23):4298. doi: 10.3390/foods12234298 (PMC10706100; doi:10.3390/foods12234298)
Supplement: Supplementary file 1 [file foods-12-04298-s001.zip › Table S3 .pdf]

Table S3 Results from the GRF1 gene ecotype Columbia (A. thaliana) sequence searches against the forward reverse and probe sequences in the NCBI (nucleotide database).

Number	Description	Accession	Blast against the NCBI GenBank		Mismatches (bp) against each accession			Similarity with the amplicon
			Query Cover	Per. ident	Forward	Reverse	Probe	
1	*Arabidopsis thaliana genome assembly, chromosome: 2*	LR782543.1	100%	100.00%	1	0	0	100%
2	*Arabidopsis thaliana genome assembly, chromosome: 2*	LR699746.2	100%	100.00%	1	0	0	100%
3	*Arabidopsis thaliana genome assembly, chromosome: 2*	LR699771.1	100%	100.00%	1	0	0	100%
4	*Arabidopsis thaliana genome assembly, chromosome: 2*	LR699766.1	100%	100.00%	1	0	0	100%
5	*Arabidopsis thaliana genome assembly, chromosome: 2*	LR699761.1	100%	100.00%	1	0	0	100%
6	*Arabidopsis thaliana genome assembly, chromosome: 2*	LR699756.1	100%	100.00%	1	0	0	100%
7	*Arabidopsis thaliana genome assembly, chromosome: 2*	LR699751.1	100%	100.00%	1	0	0	100%
8	*Arabidopsis thaliana genome assembly, chromosome: 2*	LR215053.1	100%	100.00%	1	0	0	100%
9	*Arabidopsis thaliana growth-regulating factor 1 (GRF1), mRNA*	NM_127849.4	100%	100.00%	1	0	0	100%
10	*Arabidopsis thaliana ecotype 1254 chromosome 2 sequence*	CP086755.1	100%	100.00%	1	0	0	100%
11	*Arabidopsis thaliana ecotype 5856 chromosome 2 sequence*	CP086750.1	100%	100.00%	1	0	0	100%
12	*Arabidopsis thaliana ecotype 6021 chromosome 2 sequence*	CP086745.1	100%	100.00%	1	0	0	100%
13	*Arabidopsis thaliana ecotype 6024 chromosome 2 sequence*	CP086740.1	100%	100.00%	1	0	0	100%
14	*Arabidopsis thaliana ecotype 9412 chromosome 2 sequence*	CP086735.1	100%	100.00%	1	0	0	100%
15	*Arabidopsis thaliana ecotype 9470 chromosome 2 sequence*	CP086730.1	100%	100.00%	1	0	0	100%
16	*Arabidopsis thaliana chromosome 2*	CP087127.2	100%	100.00%	1	0	0	100%
17	*Arabidopsis thaliana isolate t2t_salk_col chromosome 2*	CP096025.1	100%	100.00%	1	0	0	100%
18	*Arabidopsis thaliana genome assembly, chromosome: 2*	OW119597.1	100%	100.00%	1	0	0	100%
19	*Arabidopsis thaliana genome assembly, chromosome: 2*	LR881467.1	100%	100.00%	1	0	0	100%
20	*Arabidopsis thaliana genome assembly, chromosome: 2*	LR797808.1	100%	100.00%	1	0	0	100%
21	*Arabidopsis thaliana genome assembly, chromosome: 2*	LR797803.1	100%	100.00%	1	0	0	100%
22	*Arabidopsis thaliana genome assembly, chromosome: 2*	LR797798.1	100%	100.00%	1	0	0	100%
23	*Arabidopsis thaliana genome assembly, chromosome: 2*	LR797793.1	100%	100.00%	1	0	0	100%

24	*Arabidopsis thaliana genome assembly, chromosome: 2*	LR797788.1	100%	100.00%	1	0	0	100%
25	*Arabidopsis thaliana chromosome 2*	CP002685.1	100%	100.00%	1	0	0	100%
26	*Arabidopsis thaliana At2g22840 mRNA for hypothetical protein, partial cds, clone: RAAt2g22840*	AB493560.1	100%	100.00%	1	0	0	100%
27	*Arabidopsis thaliana isolate CS906 GRL1 (GRL1) gene, partial cds*	EU550462.1	100%	100.00%	1	0	0	100%
2829	*Arabidopsis thaliana isolate CS902 GRL1 (GRL1) gene, partial cds*	EU550456.1	100%	100.00%	1	0	0	100%
30	*Arabidopsis thaliana isolate CS6799 GRL1 (GRL1) gene, partial cds*	EU550455.1	100%	100.00%	1	0	0	100%
31	*Arabidopsis thaliana isolate CS901 GRL1 (GRL1) gene, partial cds*	EU550445.1	100%	100.00%	1	0	0	100%
32	*Arabidopsis thaliana transcription activator (GRF1) mRNA, complete cds*	AY102634.1	100%	100.00%	1	0	0	100%
33	*Arabidopsis thaliana chromosome 2 clone T20K9 map CIC06C07, complete sequence*	AC004786.3	100%	100.00%	1	0	0	100%
34	*Arabidopsis thaliana Full-length cDNA Complete sequence from clone GSLTPGH12ZD08 of Hormone Treated Callus of strain col-0 of Arabidopsis thaliana (thale cress)*	BX820248.1	100%	100.00%	1	0	0	100%
35	*PREDICTED: Arabidopsis lyrata subsp. lyrata growth-regulating factor 1 (LOC9316532), mRNA*	XM_002878592.2	100%	99.23%	2	0	0	99%
36	*PREDICTED: Camelina sativa growth-regulating factor 1-like (LOC104713726), mRNA*	XM_010430916.2	100%	99.23%	1	0	0	99%
37	*PREDICTED: Camelina sativa growth-regulating factor 1 (LOC104751923), mRNA*	XM_010473979.2	100%	99.23%	1	0	0	99%
38	*PREDICTED: Camelina sativa growth-regulating factor 1-like (LOC104704976), mRNA*	XM_010420970.1	100%	99.23%	1	0	0	99%
39	*Camelina hispida cultivar hispida voucher DAO 902780 chromosome 2*	CP094632.1	100%	98.46%	1	1	0	98%
40	*Arabidopsis arenosa genome assembly, chromosome: 4*	LR999454.1	100%	98.46%	2	1	0	98%
41	*PREDICTED: Brassica rapa growth-regulating factor 1 (LOC103858395), mRNA*	XM_009135745.3	100%	96.15%	1	1	0	96%
42	*Brassica oleracea HDEM genome, scaffold: C3*	LR031872.1	100%	96.15%	1	1	0	96%
43	*Brassica rapa genome, scaffold: A03*	LR031572.1	100%	96.15%	1	1	0	96%
44	*PREDICTED: Capsella rubella growth-regulating factor 1 (LOC17887921), mRNA*	XM_006293922.2	100%	96.15%	1	1	0	96%

45	*PREDICTED: Brassica napus growth-regulating factor 1-like (LOC125584397), mRNA*	XM_048752816.1	100%	96.15%	1	1	0	96%
46	*PREDICTED: Brassica napus growth-regulating factor 1 (LOC106389497), mRNA*	XM_013829762.3	100%	96.15%	1	1	0	96%
47	*Brassica rapa genome assembly, chromosome: A03*	LS974619.2	100%	96.15%	1	1	0	96%
48	*Brassica napus genome assembly, chromosome: C03*	HG994367.1	100%	96.15%	1	1	0	96%
49	*Brassica napus genome assembly, chromosome: A03*	HG994357.1	100%	96.15%	1	1	0	96%
50	*Brassica rapa subsp. pekinensis growth-regulating factor 1 mRNA, partial cds*	JN698986.1	100%	96.15%	1	1	0	96%
51	*PREDICTED: Brassica oleracea var. oleracea growth-regulating factor 1 (LOC106328366), mRNA*	XM_013766798.1	100%	95.38%	1	1	0	95%
52	*PREDICTED: Eutrema salsugineum growth-regulating factor 1 (LOC18021800), mRNA*	XM_006404687.2	100%	94.62%	1	2	2	95%
53	*Thlaspi arvense genome assembly, chromosome: 4*	OU466860.2	100%	94.62%	2	1	1	95%
	Raphanus sativus genome assembly, chromosome: 6	LR778315.1	98%	96.88%	1	0	0	97%
	PREDICTED: Raphanus sativus growth-regulating factor 1 (LOC108836427), mRNA	XM_018609585.1	98%	96.88%	1	0	0	97%
	Arabis alpina genome assembly, chromosome: 6	LT669793.1	93%	96.69%	1	9	2	97%
	PREDICTED: Tarenaya hassleriana growth-regulating factor 1 (LOC104807639), mRNA	XM_010533011.2	88%	88.70%	2	15	1	89%
	PREDICTED: Cucurbita pepo subsp. pepo growth-regulating factor 1-like (LOC111806015), mRNA	XM_023691334.1	87%	92.11%	2	16	0	92%
	PREDICTED: Cucurbita moschata growth-regulating factor 1-like (LOC111462890), mRNA	XM_023106693.1	87%	92.11%	2	16	0	92%
	PREDICTED: Cucurbita maxima growth-regulating factor 1-like (LOC111492063), mRNA	XM_023141237.1	87%	91.23%	2	16	0	91%
	PREDICTED: Syzygium oleosum growth-regulating factor 6-like (LOC115675732), transcript variant X2, mRNA	XM_030598485.1	86%	92.04%	2	17	0	92%
	PREDICTED: Syzygium oleosum growth-regulating factor 6-like (LOC115675732), transcript variant X1, mRNA	XM_030598484.1	86%	92.04%	2	17	0	92%
	PREDICTED: Ipomoea triloba growth-regulating factor 2-like (LOC116012194), mRNA	XM_031251676.1	86%	91.15%	2	17	0	91%

	PREDICTED: Coffea eugenioides growth-regulating factor 1-like (LOC113778176), mRNA	XM_027323475.1	86%	91.15%	2	17	0	91%
	PREDICTED: Coffea arabica growth-regulating factor 1-like (LOC113700371), transcript variant X2, mRNA	XM_027220803.1	86%	91.15%	2	17	0	91%
	PREDICTED: Coffea arabica growth-regulating factor 1-like (LOC113700371), transcript variant X1, mRNA	XM_027220802.1	86%	91.15%	2	17	0	91%
	Ipomoea triloba cultivar NCNSP0323 chromosome 3	CP025662.1	86%	91.15%	2	17	0	91%
	Ipomoea trifida cultivar NCNSP0306 chromosome 3	CP025646.1	86%	91.15%	2	17	0	91%
	PREDICTED: Coffea arabica growth-regulating factor 1-like (LOC113697940), mRNA	XM_027217565.1	86%	90.27%	2	17	1	90%
	PREDICTED: Carica papaya growth-regulating factor 1-like (LOC110809448), mRNA	XM_022035286.1	86%	90.27%	2	17	0	90%
	PREDICTED: Ipomoea nil growth-regulating factor 1-like (LOC109168875), mRNA	XM_019317731.1	86%	90.27%	2	17	0	90%
	PREDICTED: Mangifera indica growth-regulating factor 1-like (LOC123214244), transcript variant X2, mRNA	XM_044634027.1	86%	90.27%	2	17	1	90%
	PREDICTED: Mangifera indica growth-regulating factor 1-like (LOC123214244), transcript variant X1, mRNA	XM_044634025.1	86%	90.27%	2	17	1	90%
	PREDICTED: Lactuca sativa growth-regulating factor 1 (LOC111910684), mRNA	XM_023906516.2	86%	90.27%	4	17	1	90%
	PREDICTED: Nymphaea colorata growth-regulating factor 6-like (LOC116249505), mRNA	XM_031622620.1	86%	89.38%	3	17	1	89%
	PREDICTED: Elaeis guineensis growth-regulating factor 6-like (LOC105060064), mRNA	XM_010943653.3	86%	89.38%	3	17	3	89%
	PREDICTED: Camellia sinensis growth-regulating factor 1-like (LOC114276243), mRNA	XM_028218030.1	86%	89.38%	1	17	0	89%
	PREDICTED: Herrania umbratica growth-regulating factor 1 (LOC110409640), transcript variant X2, mRNA	XM_021419060.1	86%	89.38%	2	17	1	89%
	PREDICTED: Herrania umbratica growth-regulating factor 1 (LOC110409640), transcript variant X1, mRNA	XM_021419059.1	86%	89.38%	2	17	1	89%
	PREDICTED: Nicotiana attenuata growth-regulating factor 1-like (LOC109240682), mRNA	XM_019407347.1	86%	89.38%	2	17	1	89%

	PREDICTED: Rhodamnia argentea growth-regulating factor 6-like (LOC115746381), mRNA	XM_030682126.2	86%	3	3	17	1	89%
	Polygonum aviculare genome assembly, chromosome: 10	OW204032.1	86%	89.38%	2	17	0	89%
	PREDICTED: Erigeron canadensis growth-regulating factor 1-like (LOC122589483), mRNA	XM_043761777.1	86%	89.38%	4	17	0	89%
	PREDICTED: Carya illinoinensis growth-regulating factor 1-like (LOC122295810), mRNA	XM_043105073.1	86%	89.38%	1	17	0	89%
	PREDICTED: Juglans microcarpa x Juglans regia growth-regulating factor 1-like (LOC121246929), transcript variant X2, mRNA	XM_041145253.1	86%	89.38%	1	17	2	89%
	PREDICTED: Juglans microcarpa x Juglans regia growth-regulating factor 1-like (LOC121246929), transcript variant X1, mRNA	XM_041145252.1	86%	89.38%	1	17	2	89%
	PREDICTED: Eucalyptus grandis growth-regulating factor 6 (LOC104447797), mRNA	XM_010061513.3	86%	89.38%	2	17	0	89%
	PREDICTED: Helianthus annuus growth-regulating factor 1 (LOC110889297), mRNA	XM_022136801.2	86%	89.38%	4	17	1	89%
	PREDICTED: Ipomoea triloba growth-regulating factor 1-like (LOC115998161), mRNA	XM_031237652.1	86%	90.18%	2	18	0	90%
	Ipomoea triloba cultivar NCNSP0323 chromosome 12	CP025671.1	86%	90.18%	3	18	0	90%
	Ipomoea trifida cultivar NCNSP0306 chromosome 12	CP025655.1	86%	90.18%	3	18	0	90%
	PREDICTED: Ipomoea nil growth-regulating factor 2-like (LOC109177869), transcript variant X2, mRNA	XM_019327346.1	86%	90.18%	3	18	0	90%
	PREDICTED: Ipomoea nil growth-regulating factor 2-like (LOC109177869), transcript variant X1, mRNA	XM_019327345.1	86%	90.18%	3	18	0	90%
	Cucumis melo genomic scaffold, anchoredscaffold00014	LN681823.1	85%	90.09%	1	16	0	90%
	Fraxinus pennsylvanica genome assembly, chromosome: 1	OU503036.1	84%	91.82%	1	17	0	92%
	Polygonum aviculare genome assembly, chromosome: 1	OW204023.1	84%	90.00%	1	17	0	90%
	PREDICTED: Cucumis melo growth-regulating factor 6-like (LOC103487815), mRNA	XM_008446301.2	80%	91.43%	2	>20	0	91%
	Cucumis melo genomic chromosome, chr_3	LN713257.1	80%	91.43%	2	>20	0	91%
	PREDICTED: Beta vulgaris subsp. vulgaris growth-	XM_010681369.3	80%	94.23%	1	>20	0	94%

	regulating factor 2 (LOC104894980), mRNA							
	PREDICTED: Morus notabilis growth-regulating factor 1 (LOC21396183), transcript variant X2, mRNA	XM_024172566.1	80%	91.35%	2	>20	1	91%
	PREDICTED: Morus notabilis growth-regulating factor 1 (LOC21396183), transcript variant X1, mRNA	XM_010108599.2	80%	91.35%	2	>20	1	91%

No species or any threshold limitation or selection.
