# Supplementary material for: Specificity Testing for NGT PCR-Based Detection Methods in the Context of the EU GMO Regulations
Source: Foods. 2023 Nov 28;12(23):4298. doi: 10.3390/foods12234298 (PMC10706100; doi:10.3390/foods12234298)
Supplement: Supplementary file 1 [file foods-12-04298-s001.zip › Table S4.pdf]

Table S4 Results from the GRF8 gene ecotype Columbia (A. thaliana) sequence searches against the forward reverse and probe sequences in the NCBI (nucleotide database).

Description	Accession	Blast against the NCBI GenBank		Mismatches (bp) against each accession			Similarity with your amplicon
		Query Cover	Per. ident	Forward	Reverse	Probe	
Arabidopsis thaliana genome assembly, chromosome: 4	LR782545.1	100%	100%	0	3	0	100%
Arabidopsis thaliana genome assembly, chromosome: 4	LR699748.2	100%	100%	0	3	0	100%
Arabidopsis thaliana genome assembly, chromosome: 4	LR699773.1	100%	100%	0	3	0	100%
Arabidopsis thaliana genome assembly, chromosome: 4	LR699768.1	100%	100%	0	3	0	100%
Arabidopsis thaliana genome assembly, chromosome: 4	LR699758.1	100%	100%	0	3	0	100%
Arabidopsis thaliana genome assembly, chromosome: 4	LR699753.1	100%	100%	0	3	0	100%
Arabidopsis thaliana genome assembly, chromosome: 4	LR215055.1	100%	100%	0	3	0	100%
Arabidopsis thaliana ecotype 1254 chromosome 4 sequence	CP086757.1	100%	100%	0	3	0	100%
Arabidopsis thaliana ecotype 5856 chromosome 4 sequence	CP086752.1	100%	100%	0	3	0	100%
Arabidopsis thaliana ecotype 6021 chromosome 4 sequence	CP086747.1	100%	100%	0	3	0	100%
Arabidopsis thaliana ecotype 6024 chromosome 4 sequence	CP086742.1	100%	100%	0	3	0	100%
Arabidopsis thaliana ecotype 9412 chromosome 4 sequence	CP086737.1	100%	100%	0	3	0	100%
Arabidopsis thaliana ecotype 9470 chromosome 4 sequence	CP086732.1	100%	100%	0	3	0	100%
Arabidopsis thaliana chromosome 4	CP087129.2	100%	100%	0	3	0	100%
Arabidopsis thaliana isolate t2t_salk_col chromosome 4	CP096027.1	100%	100%	0	3	0	100%
Arabidopsis thaliana genome assembly, chromosome: 4	OW119599.1	100%	100%	0	3	0	100%
Arabidopsis thaliana genome assembly, chromosome: 4	LR881469.1	100%	100%	0	3	0	100%
Arabidopsis thaliana genome assembly, chromosome: 4	LR797810.1	100%	100%	0	3	0	100%
Arabidopsis thaliana genome assembly, chromosome: 4	LR797805.1	100%	100%	0	3	0	100%
Arabidopsis thaliana genome assembly, chromosome: 4	LR797800.1	100%	100%	0	3	0	100%
Arabidopsis thaliana genome assembly, chromosome: 4	LR797795.1	100%	100%	0	3	0	100%
Arabidopsis thaliana chromosome 4	CP002687.1	100%	100%	0	3	0	100%
Arabidopsis thaliana DNA chromosome 4, contig fragment No. 61	AL161561.2	100%	100%	0	3	0	100%

Arabidopsis thaliana DNA chromosome 4, BAC clone T19F6, partial sequence (ESSA project)	AL109619.1	100%	100%	0	3	0	100%
Arabidopsis thaliana chromosome IV BAC T19F6 genomic sequence, complete sequence	AC002343.1	100%	100%	0	3	0	100%
Arabidopsis thaliana genome assembly, chromosome: 4	LR699763.1	100%	97,69%	0	3	2	98%
Arabidopsis thaliana genome assembly, chromosome: 4	LR797790.1	100%	97,69%	0	3	2	98%
Arabidopsis thaliana growth-regulating factor 8 (GRF8), partial mRNA	NM_118547.2	87%	100%	0	3	>20	100%
Arabidopsis thaliana growth-regulating factor 8 (GRF8), mRNA	NM_001341645.1	30%	100%	0	3	>20	100%
Arabidopsis thaliana growth-regulating factor 8 (GRF8), partial mRNA	NM_001341644.1	30%	100%	0	3	>20	100%
Arabidopsis thaliana isolate CS902 GRL8 (GRL8) gene, partial cds	EU550550.1	25%	100%	0	3	>20	100%
Arabidopsis thaliana isolate CS901 GRL8 (GRL8) gene, partial cds	EU550539.1	25%	100%	0	3	>20	100%

No species or any threshold limitation or selection.
